# Gene Amplification in Rhabdomyosarcoma: Lessons from a Rare Cancer

**DOI:** 10.3390/ijms27052421

**Published:** 2026-03-06

**Authors:** Frederic G. Barr

**Affiliations:** Laboratory of Pathology, Center for Cancer Research, National Cancer Institute, Bethesda, MD 20892, USA; frederic.barr@nih.gov; Tel.: +1-301-480-7176; Fax: +1-301-480-0611

**Keywords:** gene amplification, alveolar rhabdomyosarcoma, *PAX3::FOXO1*, *PAX7::FOXO1*, *MYCN*, *CDK4*, *SHMT2*, *MIR17HG*

## Abstract

Studies of the pediatric soft tissue cancer alveolar rhabdomyosarcoma have contributed to the current understanding of the diverse set of molecular changes that occur as part of the gene amplification process. In accord with the traditional view of amplification, the amplicon from the 2p24 chromosomal region primarily involves a single protein-coding gene (*MYCN*). In contrast, amplification of the 12q13–q14 chromosomal region involves a gene-rich region in which there are at least two critical protein-coding oncogenic targets (*CDK4* and *SHMT2*). Amplicons involving the 1p36 and 13q14 chromosomal regions co-occur as part of a multistep process in which a mutation, in this case a translocation that forms a gene fusion (*PAX7::FOXO1*), is followed by amplification. Analysis of the amplicon involving the 13q31 region highlights an example of a situation in which the critical amplification target is a gene for a non-coding RNA (*MIR17HG*) instead of a protein-coding gene. Translational studies of the prognostic significance of these amplicons emphasize important considerations encountered in defining useful prognostic markers. Finally, preclinical investigations revealed that some amplification events (*CDK4* and *SHMT2*) decrease susceptibility to drugs that directly target the amplified gene products and increase susceptibility to drugs targeting proteins that function in signaling pathways downstream of these amplified gene products. These combined studies in alveolar rhabdomyosarcoma emphasize the biological and clinical complexities of gene amplification in cancer.

## 1. Introduction

**Figure 1:**
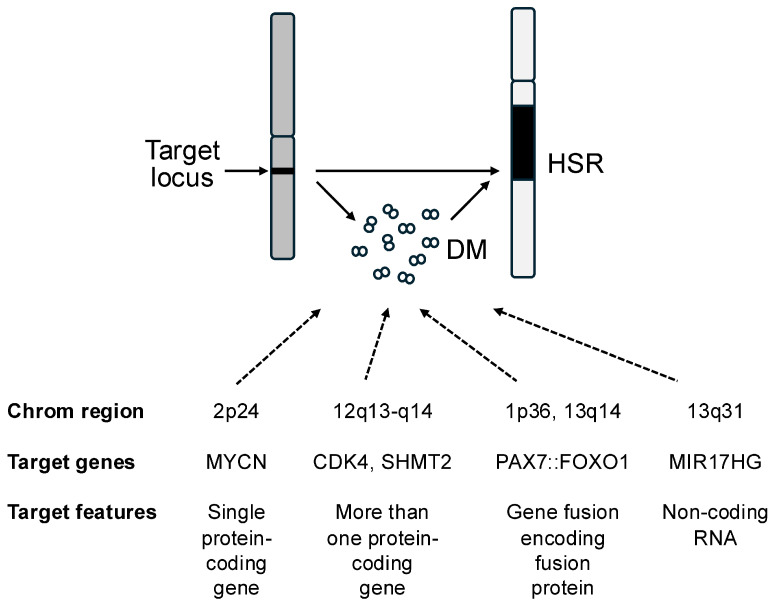
Overview of the common amplification events in ARMS. Amplification can be detected by the presence of double minutes (DMs) and/or a HSR (homogeneously staining region). The four common amplification events in ARMS are shown.

Gene amplification is one of the classic genetic mechanisms for activating oncogenes and aberrantly stimulating pathways involved in growth and other oncogenic activities [1,2]. In the amplification process, the copy number of an oncogene and the surrounding genomic region is aberrantly increased above the usual two copies per cell, often to much higher levels, which can be as high as several hundred-fold. In contrast to polysomy where there is whole chromosome gain, amplification specifically refers to the setting where there is an increased copy number of a focal chromosomal region. The amplified segments are usually present as extrachromosomal elements (double minutes) or a tandem array integrated into one of the chromosomes (homogeneously staining region) (Figure 1). Two well-characterized amplification events involve the *MYCN* gene from the 2p24 chromosomal region in neuroblastoma and the *ERBB2* gene from the 17q12–q21 chromosomal region in breast carcinoma. Based on initial studies of these and other amplicons, the traditional view focused on a single wild-type protein-encoding gene within the amplified region as the oncogenic target of the amplification. However, subsequent studies expanded this view and revealed a diversity of underlying molecular changes and clinical considerations. In this review, the molecular diversity and clinical significance of amplification events will be illustrated by consideration of the amplicons occurring in the soft tissue cancer alveolar rhabdomyosarcoma (ARMS).

## 2. Alveolar Rhabdomyosarcoma

Rhabdomyosarcoma (RMS) is a family of soft tissue cancers that are associated with the myogenic lineage and often occur in the pediatric population [3,4]. The alveolar subtype was initially identified based on a characteristic microscopic morphology of small round cell aggregates in which clefts form that resemble alveolar spaces in the lung. Subsequent cytogenetic and molecular studies of ARMS cases identified recurrent chromosomal translocation events that result in gene fusions encoding fusion oncoproteins [5]. The most common translocation event in ARMS juxtaposes the 2q35 and 13q14 chromosomal regions to generate a *PAX3::FOXO1* (*P3F*) fusion, and a less frequent variant translocation event juxtaposes the 1p36 and 13q14 regions to generate a *PAX7::FOXO1* (*P7F*) fusion. These fusions combine functional domains from PAX and FOXO proteins and encode novel chimeric transcription factors. RT-PCR assays of numerous ARMS cases demonstrated that the *P3F* and *P7F* fusions occur in approximately 60% and 20%, respectively, of ARMS cases [4,6]. Rare variant fusions were identified in a small subset of the remaining 20% but most of these cases do not contain any fusions and represent a true fusion-negative category of ARMS tumors, which has genetic and clinical features similar to the embryonal subtype of RMS [3,7].

To determine whether there are other genetic changes in ARMS that may collaborate with the gene fusions, a number of genome-wide methodologies, including comparative genomic hybridization, microarrays and next-generation sequencing, were applied to cohorts of ARMS cases [8,9,10,11,12]. Although recurrent point mutations are uncommon in ARMS, amplification events were identified in more than half of fusion-positive ARMS cases. Furthermore, five genomic regions are frequently amplified in ARMS and correspond to the 1p36, 2p24, 12q13–q14, 13q14 and 13q31 chromosomal regions [13,14,15]. These amplification events are generally confined to ARMS tumors with the *P3F* or *P7F* fusion, which supports the postulate that fusion-negative ARMS is more similar from a genetic and biologic perspective to fusion-negative embryonal RMS. Within the fusion-positive ARMS category, the amplicons often have a preference for either the *P3F* or the *P7F* subset (Table 1). In particular, the 12q13–q14 amplicon preferentially occurs within the *P3F* subset whereas the 1p36, 13q14 and 13q31 amplicons preferentially occur within the *P7F* subset. Finally, the 2p24 amplicon occurs in a similar fraction of both *P3F* and *P7F* subsets.

## 3. Single Protein-Encoding Target Gene Within an Amplicon—2p24 Amplicon in ARMS

The finding of frequent amplification of the 2p24 chromosomal region in ARMS suggested that *MYCN* would be amplified similarly to the findings in neuroblastoma [9,12,13]. Using DNA microarray data for five ARMS cases with 2p24 amplification, a minimal common region of amplification was defined in which there were two protein-encoding genes [13]. *MYCN* is located within the center of the minimal common region whereas *DDX1* is located at the periphery of this region. Quantitative RT-PCR studies of a larger cohort of RMS cases, including 15 cases with and 91 cases without 2p24 amplification, showed that *MYCN* and *DDX1* are expressed at higher levels in 2p24-amplified compared to non-amplified cases. However, there is a subset of amplified cases in which *MYCN* expression is high but *DDX1* expression is low, thus indicating that *MYCN* is more consistently overexpressed in the 2p24-amplified cases. A likely explanation of these findings is that *DDX1* is not amplified in all ARMS cases with 2p24 amplification, similarly to the findings in neuroblastoma [16].

Numerous studies have demonstrated that Myc family proteins, including MYC and MYCN, have oncogenic effects in both cell culture and animal assays [17,18]. However, only a few studies have specifically examined the role of MYCN in fusion-positive ARMS. RNA interference or antigene peptide nucleic acid approaches inhibit *MYCN* expression in ARMS cell lines and result in reduced proliferation and increased apoptosis [19]. To examine the effect of MYCN on P3F oncogenic activity, *MYCN* and/or *P3F* expression constructs were transduced into human myoblast cell culture systems [20,21,22]. Neither P3F nor MYCN is capable of inducing oncogenic transformation alone in these systems, but the combination of P3F and MYCN induces transformation, thus suggesting that MYCN directly collaborates with P3F. These combined studies thus provide evidence supporting an oncogenic role for *MYCN* amplification in ARMS.

## 4. Multiple Target Genes Within an Amplicon—12q13–q14 Amplicon in ARMS

For the amplification events involving the 12q13–q14 region, an alternative approach was used to localize the commonly amplified genomic loci in ARMS [23]. In a statistical approach, the 12q13–q14 region was subdivided into multiple segments and microarray data for the probes in each segment were used to determine the segments in which there were statistically significant differences in copy number between amplified and non-amplified ARMS cases. This analysis revealed a high-confidence region of 12q13–q14 amplification in fusion-positive ARMS measuring 0.8 Mb. This region is similar to the minimal common region of 12q13–q14 amplification identified in an earlier study [13]. Extrapolation of this commonly amplified segment on the genetic map demonstrates that this amplification involves a gene-rich region in which over 30 genes are commonly amplified [23]. RNA-seq analysis of the expression levels of genes in this region revealed that only 14 genes from this 0.8 Mb segment show statistically significant differences in expression between amplified and non-amplified ARMS cases. This finding emphasizes that an increase in a gene’s copy number does not necessarily result in increased expression of that gene, and thus only a subset of genes in an amplified segment may contribute to the functional effect of that amplification event.

Amplification of the 12q13–q14 region occurs in other cancer categories, such as glioblastoma multiforme (GBM), dedifferentiated liposarcoma (DDLPS) and lung adenocarcinoma (LUAD) [23]. The statistical mapping approach described above was applied to these other cancer categories and showed that the high-confidence regions of amplification vary among these categories. In particular, the size of the commonly amplified region varies from 0.5 Mb in GBM to over 1 Mb in LUAD, but all four categories share a 0.2 Mb region of overlap. Beyond this region of overlap, there is variability in the extent of the regions that are commonly amplified in each cancer category. The commonly amplified region in ARMS extends the furthest towards the centromere, resulting in a 0.5 Mb region that is specifically amplified in ARMS. Based on these results, the commonly amplified region in ARMS can be divided into subregions, including the 0.2 Mb overlap region and the 0.5 Mb ARMS-specific region. The 0.2 Mb overlap region contains 15 genes, of which seven are expressed at higher levels in tumors with 12q13–q14 amplicons from all four cancer categories (*OS9*, *TSPAN31*, *CDK4*, *CYP27B1*, *METTL1*, *EEF1AKMT3*, and *TSFM*). The 0.5 Mb ARMS-specific region contains 18 genes, of which only four show increased expression in ARMS tumors with 12q13–q14 amplicons (*NEMP1*, *NAB2*, *SHMT2*, and *R3HDM2*).

Although the oncogenic function of amplified/overexpressed genes from the overlap and ARMS-specific regions has not been fully explored, oncogenic activity has been demonstrated for one gene from each region, *CDK4* and *SHMT2*. *CDK4* encodes a kinase that stimulates RB-E2F signaling and promotes cell cycle progression from G1 to S phase [24]. Previous studies showed that *CDK4* overexpression or mutation enhances tumorigenesis, thus establishing *CDK4* as an oncogene. In an ARMS cell line with 12q13–q14 amplification, shRNA-induced depletion of CDK4 results in decreased expression of E2F target genes along with decreases in proliferation, oncogenic transformation and in vivo tumor growth [25]. The significance of this finding must be tempered by the finding that CDK4 depletion in an ARMS line without 12q13–q14 amplification also inhibited proliferation and oncogenic transformation; these findings are consistent with the premise that CDK4 function is critical in cells with or without *CDK4* amplification. In a complementary study, CDK4 overexpression in an ARMS line without 12q13–q14 amplification did not detectably increase expression of E2F target genes, proliferation or oncogenic transformation, thus indicating that CDK4 overexpression is not sufficient to drive a functional change in these ARMS cells. A possible explanation of this finding is that other pre-existing molecular changes may already stimulate RB-E2F signaling in these non-amplified RMS cells, and thus the functional effect of CDK4 overexpression may be best observed in non-transformed cell types without pre-existing changes in RB-E2F signaling.

The *SHMT2* gene within the ARMS-specific amplified region encodes a mitochondrial enzyme that functions in the conversion of glycine to serine and generates one-carbon intermediates that contribute to the synthesis of numerous essential molecules, such as pyrimidines and purines [26]. In the ARMS cell line with 12q13–q14 amplification, shRNA-induced depletion of SHMT2 resulted in reduced NADPH levels along with decreased proliferation, oncogenic transformation and in vivo tumor growth [23]. Similar to the *CDK4* studies cited above, SHMT2 depletion in ARMS lines without 12q13–q14 amplification also inhibited proliferation and transformation, suggesting that baseline SHMT2 function is critical and independent of amplification status. However, the complementary analysis of SHMT2 overexpression revealed increased NADPH levels along with increased proliferation, oncogenic transformation and in vivo tumor growth, thus confirming that *SHMT2* amplification contributes to oncogenesis.

## 5. Amplification of Fusion Genes—1p36 and 13q14 Amplicons in ARMS

DNA microarray studies of ARMS tumors determined that amplicons originating from the 1p36 and 13q14 chromosomal regions usually occur in the same tumors [14]. The minimal common region of 1p36 amplification contains the 5′ exons (but not the 3′ exons) of the *PAX7* gene and the minimal common region of 13q14 amplification contains the 3′ exons (but not the 5′ exons) of the *FOXO1* gene. These findings are consistent with the premise that the fusion gene consisting of the 5′ portion of *PAX7* and the 3′ portion of *FOXO1* is amplified in these ARMS tumors. This hypothesis is validated by FISH analysis, with probes specific for *PAX7* and *FOXO1* revealing multiple copies of the *P7F* fusion gene [27]. Subsequent FISH analysis of numerous ARMS cases demonstrates that this amplification process occurs in >90% of ARMS cases with the *P7F* fusion [14]. These findings suggest that a two-step process in ARMS tumorigenesis consists of a first step in which the 1;13 chromosomal translocation forms the *P7F* fusion gene followed by a second amplification step that increases the copy number of a focal segment containing this fusion gene.

Gene expression studies of *P7F*-positive tumors revealed that this amplification process is associated with a higher level of the *P7F* fusion transcript compared to the transcript arising from the non-amplified wild-type *PAX7* gene [14]. Of note, amplification of the *P3F* fusion gene is much less common and occurs in <10% of *P3F*-posistive ARMS cases. Despite the lack of *P3F* amplification, there is a higher level of the *P3F* fusion transcript compared to the wild-type *PAX3* transcript in *P3F*-positive ARMS tumors due to a copy number-independent increase in transcription [28]. A direct comparison of the fusion mRNA levels in the two fusion subsets indicates that the *P7F* levels are approximately four-fold higher than the *P3F* levels [14]. The molecular differences between *P3F* and *P7F* that give rise to these differing molecular mechanisms for fusion gene overexpression are not clear but may relate to differences between the *PAX3* and *PAX7* gene promoters [29]. In both fusion subsets, the common overexpression endpoint suggests an obligate need for high-level fusion gene expression to surpass some oncogenic threshold during ARMS tumorigenesis.

## 6. Amplification of Genes for Non-Coding RNA—13q31 Amplicon in ARMS

For the amplicon in ARMS derived from the 13q31 chromosomal region [9], the minimal common region of amplification was localized to a 0.15 Mb segment [15]. By superimposing the genetic map of this region, it was shown that the minimal common region of amplification does not contain any protein-coding genes and in particular lacks the nearby *GPC5* gene. Instead, this minimal common region only contains the *MIR17HG* gene and a peptidylprolyl isomerase pseudogene *LOC390419*. Whereas the spliced *MIR17HG* RNA product is a long non-coding RNA, the third intron of *MIR17HG* contains a region from which the miR-17-92 cluster of microRNAs is transcribed [30]. This miR-17-92 cluster contains six microRNAs (miRNAs), which are short (18–22 nt) non-coding RNAs that regulate expression of protein-encoding genes by decreasing mRNA stability and/or inhibiting translation of the polypeptide product.

Gene expression studies showed that *MIR17HG* amplification results in increased expression of the MIR17HG long non-coding RNA as well as increased expression of five of the six miRNAs of the miR-17-92 cluster [15]. The one miRNA within this cluster that was not upregulated is miR-18a, which appears to be processed by a different mechanism than the other five miRNAs. Studies in other cancers have shown that the targets for miRNAs of the miR-17-92 cluster include multiple tumor suppressor genes, such as the genes encoding the cyclin-dependent kinase inhibitor p21 and the proapoptotic protein Bim [30]. It is therefore hypothesized that *MIR17HG* amplification results in higher levels of five of the miRNAs from the miR-17-92 cluster, which in turn results in lower levels of the targeted tumor suppressor proteins. Available evidence supports the premise that elevated expression of the miRNAs of the miR-17-92 cluster stimulates oncogenic behavior and thus these miRNAs are termed “oncomirs”.

## 7. Clinical Significance of Amplification in ARMS

Several studies have investigated the prognostic significance of the amplicons in ARMS (Table 2). In particular, multiple studies focused on the *MYCN*-containing amplicon from the 2p24 region and reached differing conclusions [13,31,32,33]. Whereas the largest study of 93 cases did not identify any significant difference in outcome between cases with and without *MYCN* amplification [13], several smaller studies found evidence that *MYCN*-amplified cases have a significantly worse outcome [31,32,33]. The explanation for these differing findings can be attributed to various factors, including the small size of the cohorts and even smaller number of amplified cases, differing technical methodology and quantitative criteria for amplification, and varying distributions of treatment regimens. In addition, as fusion-negative ARMS cases do not generally have these amplicons but have a better outcome than fusion-positive ARMS cases [7], the inclusion of fusion-negative ARMS cases can influence the prognostic effect determined in these studies.

For assessing prognostic significance of the *CDK4*-containing amplicon from the 12q13–q14 region, two relatively large studies found evidence that this amplicon was associated with a significantly worse outcome [13,32]. Since this amplicon is mostly found in *P3F*-positive cases, an analysis was also performed exclusively in the *P3F*-positive category (*n* = 45) and confirmed that this amplicon is associated with a significant decrease in failure-free survival (*p* = 0.042) and a marked trend toward a worse overall survival (*p* = 0.056) [13]. To determine if this amplicon is a significant independent predictor of outcome, a multivariate analysis of amplification status along with clinical group, tumor size, lymph node status, and primary site was conducted and determined that, after adjusting for the effects of the other clinical variables, the *CDK4*-containing amplicon was not independently predictive of failure-free survival (*p* = 0.19) or overall survival (*p* = 0.30).

Only single clinical correlative studies were conducted to examine the *FOXO1*- and *MIR17HG*-containing amplicons from the 13q14 and 13q31 regions, respectively [14,15]. In each case, these studies (86–87 cases) showed that these amplicons were significantly associated with improved overall survival and a trend towards improved event-free survival. However, these results must be further interpreted since the *P7F* and *MIR17HG* genes are amplified in >90% and 75%, respectively, of *P7F*-positive cases. Based on evidence that *P7F*-positive cases have a more favorable outcome than *P3F*-positive cases [14,34,35], the *P7F*- or *MIR17HG*-amplified cases may start out with a more favorable outcome. There is a need to focus on the *P7F*-positive subset and compare the outcome of amplified and non-amplified cases within this RMS subpopulation. In the studies to date, the number of available non-amplified *P7F* cases is inadequate to examine this question and thus the effects on outcome cannot be definitively compared.

## 8. Targeted Therapy of Tumors with Amplicons

As was first shown for *ERBB2*-amplified breast carcinomas, there have been efforts to identify drugs that bind to and inhibit function of overexpressed proteins encoded by amplified genes. Such drugs may serve as a targeted therapy for tumors in which one of these amplification events occurs. To date, efforts in ARMS have focused on amplification of the 12q13–q14 chromosomal region [23,25]. After several drugs were developed that bind and inactivate CDK4 (and the related CDK6 protein), *CDK4* amplification was postulated to sensitize ARMS cells to these inhibitors. However, studies of the available ARMS cell lines revealed that Rh30 cells, which have *CDK4* amplification, are relatively insensitive, both in vitro and in vivo, to the CDK4 inhibitor ribociclib (LEEO11) compared to other ARMS lines without this amplicon [25]. Furthermore, engineered inducible CDK4 expression in the Rh28 ARMS line, which has low endogenous CDK4 expression, resulted in decreased sensitivity to ribociclib. Similarly, treatment of ARMS lines with SHIN1, a drug that directly targets and inhibits SHMT2, also showed low SHIN1 sensitivity in Rh30 cells [23]. Decreased SHMT2 expression (via shRNA) in Rh30 cells resulted in increased sensitivity to SHIN1 whereas increased SHMT2 expression in non-amplified ARMS lines resulted in decreased sensitivity to SHIN1. These combined results provide evidence of a setting in which tumor cells with oncogene amplification are relatively insensitive to therapy targeting the corresponding oncoprotein. Such an effect is similar to the methotrexate resistance seen in acute leukemias with *DHFR* amplification, which was proposed to increase DHFR protein expression and thereby decrease potency of the direct inhibitor by binding and sequestering the drug [36].

The SHMT2 protein functions within a metabolic pathway that adds one carbon unit to tetrahydrofolate to provide building blocks for downstream purine and pyrimidine synthesis [37]. Increased SHMT2 protein expression from gene amplification results in increased production of downstream folate metabolites without any change in expression of the downstream metabolic enzymes. Since an increased SHMT2 protein level may sequester the direct-acting drug SHIN1, experiments were conducted to assess whether a drug targeting a downstream metabolic enzyme may be more effective [23]. The folate analog pemetrexed, which inhibits enzymes involved in purine and pyrimidine synthesis, was selected as an indirect-acting drug for these experiments. In contrast to the results with the direct inhibitor SHIN1, treatment of ARMS lines with pemetrexed shows the highest sensitivity in Rh30 cells, which have *SHMT2* amplification. Furthermore, shRNA-induced SHMT2 downregulation in Rh30 cells resulted in decreased pemetrexed sensitivity whereas increased SHMT2 expression in non-amplified ARMS lines resulted in increased pemetrexed sensitivity. These results thus present a therapeutic strategy for bypassing the sequestration effect associated with amplification-induced oncoprotein overexpression. For the potential application of pemetrexed in *SHMT2*-amplified ARMS, studies are next needed to determine the in vivo efficacy of this therapeutic strategy.

## 9. Discussion and Conclusions

Initial studies of gene amplification in cancer focused on the scenario of a wild-type protein-encoding gene as the oncogenic target of the cancer-associated amplification event. However, more recent studies of other amplification events, such as the ARMS studies described above, expanded this narrow view of cancer amplicons and revealed that amplification can be associated with a diverse set of molecular changes (Figure 1). In particular, studies of the 12q13–q14 amplicon showed that there may be multiple targets within an amplicon. Studies of the 1p36 and 13q14 amplicons highlighted that the amplification target may be a mutated gene, in this case a fusion gene resulting from a translocation event. Finally, studies of the 13q31 amplicon revealed that a gene for a non-coding RNA may be the critical amplification target instead of a protein-coding gene.

In addition to these biological issues, translational studies in ARMS emphasize the challenges in establishing the clinical utility of amplification events and employing these events as biomarkers in risk stratification. Determination of the prognostic significance of an amplification event requires large, well-annotated cohorts in which variables such as fusion status or treatment can be controlled. Even when prognostic significance is established in a univariate study, multivariate studies are needed to determine whether the amplicon has independent predictive value or whether its predictive significance is subsumed by one or more previously known prognostic markers. Finally, these studies in ARMS have relevance to treatment considerations as they point out that an amplification event may not enhance sensitivity to a drug that directly targets an overexpressed protein encoded within the amplicon. Instead, the amplicon may actually decrease sensitivity to that drug due to sequestration by an overexpressed target protein. In such instances, as exemplified by the studies of pemetrexed in *SHMT2* amplification, an alternative targeted therapeutic approach may be instituted in which drugs are selected to target proteins that function downstream in signaling pathways activated by the amplification event.

In the future, it is anticipated that therapeutic approaches will be devised to directly or indirectly target other recurrent amplification events. These amplicon-specific therapies may be applicable to multiple cancer categories and permit a tissue-agnostic “bucket” approach. In addition, recent studies of extra-chromosomal DNA (ecDNA) produced by amplification events identified cellular processes required for the maintenance of these abnormal genomic features and suggest that therapeutic approaches may be able to exploit potential vulnerabilities associated with these cellular processes [38]. For example, the high replication stress associated with ecDNA maintenance is managed by signaling pathways involving S-phase checkpoint mediators such as CHK1 kinase. The therapeutic potential of this approach is suggested by the finding that ecDNA-containing cells are sensitive to CHK1 inhibitors, particularly when combined with an inhibitor targeting the relevant amplicon.

It should be emphasized that these amplicons are generally not considered to be initiating events but are rather viewed as critical collaborating events that modify clinical and biological phenotype. In ARMS, the gene fusions are the likely initiating events, and substantial effort has also been invested to explore strategies that antagonize expression or function of these fusion oncoproteins and downstream transcriptional targets [39]. Finally, epigenetic studies have revealed increased or decreased expression of other molecular features in ARMS cells that contribute to the biology of this tumor and its constellation of signaling pathways [40,41]. This combination of gene fusions, amplification events and other epigenetic changes will hopefully provide a large number of potential targets for future therapeutic advances.

## Figures and Tables

**Table 1:** Major amplicons in alveolar rhabdomyosarcoma.

Amplicon	1p36	2p24	12q13–q14	13q14	13q31
Target Oncogene	5′ *PAX7*	*MYCN*	*CDK4* *SHMT2*	3′ *FOXO1*	*MIR17HG*
Incidence					
*PAX3::FOXO1*	0%	18%	24%	9%	8%
*PAX7::FOXO1*	89%	22%	4%	93%	75%
Fusion-negative	0%	2%	0%	0%	5%
Other cancers	---	NB	GBM LUAD LPS	---	BCL

Abbreviations: NB, neuroblastoma; GBM, glioblastoma multiforme; LUAD, lung adenocarcinoma; LPS, liposarcoma; BCL, B-cell lymphoma.

**Table 2:** Clinical outcome association with specific amplicons in ARMS.

Gene	Method	Cut-Off	Category	Amp Freq	FFS	OS	Effect	Ref.
*MYCN*	FISH	*MYCN* > control *	ARMS	9/15 (60%)	NA	*p* < 0.05	Worse	[31]
	qPCR	*MYCN* > 1.5× control	ARMS	10/38 (26%)	*p* = 0.041	*p* = 0.021	Worse	[33]
	FISH	20 copies	ARMS	10/93 (11%)	*p* = 0.57	*p* = 0.87	None	[13]
	NGS	6 copies	ARMS and/or FP	14/69 (20%)	*p* = 0.045	*p* = 0.29	Worse	[32]
	NGS	6 copies	ARMS and/or FP	2/57 (4%)	*p* = 0.44	*p* = 0.15	None	[32]
*CDK4*	FISH	10 copies	ARMS	11/92 (12%)	*p* = 0.023	*p* = 0.04	Worse	[13]
	NGS	6 copies	ARMS and/or FP	10/69 (14%)	*p* = 0.045	*p* = 0.29	Worse	[32]
	NGS	6 copies	ARMS and/or FP	3/57 (5%)	*p* = 0.44	*p* = 0.15	None	[32]
*MIR17HG*	FISH	*MYCN* > 5× control	ARMS	18/86 (21%)	*p* = 0.085	*p* = 0.013	Better	[15]
*FOXO1*	FISH	10 copies	FP RMS	28/87 (32%)	*p* = 0.053	*p* = 0.001	Better	[14]

* The control probe was from a locus in another region of chromosome 2. Abbreviations: FISH, fluorescence in situ hybridization; qPCR, quantitative polymerase chain reaction; NGS, next-generation sequencing; ARMS, alveolar rhabdomyosarcoma; FP, fusion-positive; RMS, rhabdomyosarcoma; Amp Freq, amplification frequency; FFS, failure-free survival; NA, not available; OS, overall survival.

## Data Availability

No new data were created or analyzed in this study. Data sharing is not applicable to this article.
